# FGF10 and Human Lung Disease Across the Life Spectrum

**DOI:** 10.3389/fgene.2018.00517

**Published:** 2018-10-31

**Authors:** Lawrence S. Prince

**Affiliations:** Department of Pediatrics, University of California, San Diego, Rady Children’s Hospital, San Diego, CA, United States

**Keywords:** branching morphogenesis, alveolar epithelia, lung injury, inflammation, lung regeneration and repair

## Abstract

Lung diseases impact patients across the lifespan, from infants in the first minutes of life through the aged population. Congenital abnormalities of lung structure can cause lung disease at birth or make adults more susceptible to chronic disease. Continuous inhalation of atmospheric components also requires the lung to be resilient to cellular injury. Fibroblast growth factor 10 (FGF10) regulates multiple stages of structural lung morphogenesis, cellular differentiation, and the response to injury. As a driver of lung airway branching morphogenesis, FGF10 signaling defects during development lead to neonatal lung disease. Alternatively, congenital airway abnormalities attributed to FGF10 mutations increase the risk of chronic airway disease in adulthood. FGF10 also maintains progenitor cell populations in the airway and promotes alveolar type 2 cell expansion and differentiation following injury. Here we review the cellular and molecular mechanisms linking FGF10 to multiple lung diseases, from bronchopulmonary dysplasia in extremely preterm neonates, cystic fibrosis in children, and chronic adult lung disorders. Understanding the connections between FGF10 and lung diseases may lead to exciting new therapeutic strategies.

## Introduction

Lung structure and physiology has remained consistent throughout mammalian evolution (Mess and Ferner, 2010). With a >100 m^2^ surface area available for gas exchange (Colebatch and Ng, 1992), human lungs efficiently deliver oxygen to the circulation and expel carbon dioxide produced by aerobic cellular respiration. The lung can be separated into two distinct regions based on physiological function. Conducting airways begin just below the larynx and trachea. Stereotypical branches form 23 generations in human lungs (Weibel and Gomez, 1962) and 10 generations in mice (Kizhakke Puliyakote et al., 2016) of smaller and smaller airways. The trachea, bronchi, and bronchioles serve to warm and humidify inhaled air from the environment. In addition, airway mucous captures inhaled particulates, allowing efficient removal by mucociliary clearance (Munkholm and Mortensen, 2014). Multiciliated epithelia move aggregates of mucous and its sequestered material upon a thin layer of airway surface liquid in a cranial direction for expectoration or ingestion. The movement and filtering of inhaled gas through the conducting airways delivers clean, warm air to the alveolar structures for oxygen and carbon dioxide exchange.

The alveolar surface is lined with two types of epithelial cells (Guillot et al., 2013). Alveolar type 1 (AT1) cells are flat, thin epithelia that cover the vast majority of the alveolar surface. AT1 cells reside in intimate proximity with underlying alveolar capillary vascular endothelial cells, permitting efficient oxygen uptake and carbon dioxide removal. AT1 and endothelial cells rely upon mechanical support from extracellular elastic fibers and a variety of mesenchymal-derived interstitial lung fibroblasts (Pierce et al., 1995; Starcher, 2000). Alveolar type 2 (AT2) cells are cuboidal epithelial cells responsible for the production of lung surfactant (Veldhuizen et al., 2000). The combination of amphipathic surfactant proteins and lipids form organized structures along the alveolar surface termed tubular myelin. As intra-alveolar pressures change with each breath, folding and unfolding of the tubular myelin maintains alveolar size by modulating surface tension (Johansson et al., 1994). In addition to the physiological roles of delivering oxygen to the systemic circulation and removing carbon dioxide, each of the cell populations in the lung play important roles in protecting against infection and repairing the lung following mechanical, chemical, or biological damage (Barkauskas et al., 2013; Chambers and Mercer, 2015; Wang et al., 2016).

## FGF10 and Lung Morphogenesis

Formation of the unique structures within the lung involves complex molecular and cellular processes. Fibroblast growth factors (FGFs) play important roles throughout lung morphogenesis. Twenty-two different FGF family members have been identified and characterized, with seven subfamilies based on protein structure similarities (Ornitz and Itoh, 2015). FGF10 (KGF2) is a member of the FGF7 subfamily and critical for lung morphogenesis, cellular differentiation, and repair following injury. Produced primarily by mesenchymal populations in the lung interstitium, secreted FGF10 tightly binds heparin sulfate proteoglycans (HSPG) in the extracellular matrix. HSPG interactions stabilize FGF10, restrict its diffusion, and facilitate binding to FGF receptors (Makarenkova et al., 2009). FGF10 binds both FGFR1 and FGFR2, with highest affinity for the alternatively spliced FGFR2IIIb isoform (Lu et al., 1999). Formation of symmetrical FGF10, HSPG, and FGFR2 dimers leads to receptor activation of downstream receptor tyrosine kinase targets (Schlessinger et al., 2000).

During the earliest stages of lung development, FGF10 is expressed in the mesenchyme surrounding the distal tips of branching airways (Bellusci et al., 1997). Retinoic acid and Wnt ligands stimulate early FGF10 expression (Desai et al., 2004; Li et al., 2005). Epithelial cells in the branching airways express the receptor FGFR2, establishing a paracrine signaling system. Knockout mice studies have demonstrated the critical importance of FGF10 in lung morphogenesis. Mice lacking FGF10 form an embryonic trachea, but only two rudimentary buds marking where the right and left mainstem bronchi should normally develop (Sekine et al., 1999). Conditional mouse models and *ex vivo* studies have illustrated how FGF10 promotes later stages of lung morphogenesis. FGF10 stimulates migration of the leading edge of newly developing airways. Elongation of these tubular, epithelial-lined structures involves specific orientation of FGF-stimulated, dividing epithelial cells (Tang et al., 2011). Unlike some other branching epithelial organs, lung airways maintain a simple epithelial orientation with nearly continuous contact with luminal lung fluid.

Along with promoting airway elongation, FGF10 induces expression of epithelial factors that negatively regulate FGF10 expression. Activated FGFR2 signals through ETS-related transcription factors Etv4 and Etv5 (Herriges et al., 2015). In the branching embryonic lung, FGF10 induces epithelial expression of Bmp4 and Shh at the leading airway edge. In a coordinated negative feedback loop, both Bmp4 and Shh reduce expression of FGF10 in the adjacent mesenchyme. As FGF10 expression distal to the leading airway falls, a branch point is established, generating a structural split of the airway and subsequent elongation toward more lateral locations where FGF10 expression has not yet been inhibited. Multiple iterations of the process generate the stereotypical pattern of conducting airways unique to each mammalian species (Miura, 2008; Celliere et al., 2012). Bmp4 also promotes mesenchymal differentiation of peribronchial smooth muscle and slows epithelial proliferation, both of which could regulate the branching process (Kim and Vu, 2006). Precise regulation of FGF10 expression is critical for normal morphogenesis, as overexpression of FGF10 during development leads to cystic adenomatoid malformations (Gonzaga et al., 2008).

After conducting airway branching completes, formation of saccular airways occurs via presumably random branching and division of distal airway structures. Saccular airways become alveolar ducts later in development and the number of terminal saccular airways likely determine the eventual number of alveolar units in the mature lung (Burri, 1984). The mechanisms of saccular branching share many attributes with conducting airway branching, including the role of FGF10 in airway elongation and branching (Benjamin et al., 2007). The branching process ceases as saccular airway epithelia begin differentiating into AT1 and AT2 cells.

FGF10 also drives formation of normal alveolar structures later in lung development. Following completion of distal airway branching at the end of the saccular stage, alveolar formation produces the mature lung structures capable of efficient gas exchange. Generation of mature alveoli involves the division of distal airspaces into smaller structures, increasing the effective surface area for gas exchange. Alveolar division or septation requires mechanical forces generated by *Acta2*-positive alveolar myofibroblasts within the lung mesenchyme (Branchfield et al., 2016). Myofibroblasts arise from *Pdgfra*-positive mesenchymal cells, which express FGF10 and often have lipofibroblast characteristics. Mice with reduced FGF10 expression have fewer *Acta2*-positive myofibroblasts at birth and fail to form normal alveolar structures (Ramasamy et al., 2007). Overexpression of a dominant negative, soluble *Fgfr* transgene also reduced alveolar formation. In a pneumonectomy model of alveolar regeneration, interfering with FGFR2 signaling prevented myofibroblast differentiation (Chen et al., 2012). These observations could involve autocrine mechanisms where FGF ligands including FGF10 directly regulate fibroblast phenotype or paracrine signaling loops involving Shh, Wnt, Bmp, or TGFβ signaling between adjacent cell populations.

In addition to driving structural morphogenesis of conducting airways and alveoli, FGF10 also regulates lung cell differentiation. Epithelia lining the trachea and large airways contain multipotent progenitor basal cells – sometimes referred to as basal stem cells (Pardo-Saganta et al., 2015). Mesenchymal FGF10 expression in the trachea establishes a unique niche for basal cells, with FGF10 driving basal cell expansion and preventing terminal epithelial differentiation. Interestingly, FGF10 expression in the peri-tracheal mesenchyme is restricted to areas between cartilaginous rings (Sala et al., 2011). Within the tracheal epithelium, downregulation of Hippo activity in basal cells results in nuclear Yap localization and Wnt7b expression (Volckaert et al., 2017). Epithelial Wnt7b then increases or at least maintains FGF10 expression in the underlying mesenchyme. In smaller distal airways, the lack of FGF10 expression in peribronchial smooth muscle cells correlates with epithelial differentiation and reduced basal cell number. However inactivation of Hippo in smaller airways increases FGF10 expression in smooth muscle and leads to ectopic basal cell expansion. This highly regulated basal cell niche beautifully illustrates the paracrine nature of FGF10 signaling in maintaining normal lung biology throughout the lifespan.

FGF10 is also important for AT2 cell differentiation. As fetal saccular airways complete branching, elongation, and expansion, alveolar epithelial differentiation begins proximal to the airway leading edge. As the distal air saccules expand with fetal lung fluid, cuboidal cells are observed protruding from the epithelial monolayer in a basal direction (Li et al., 2018). These protruding cells differentiate into AT2 cells, while cells remaining in the epithelial monolayer are subjected to increased stretch and acquire an AT1 phenotype. Increasing airway distension promotes AT1 differentiation, while reducing stretch increases AT2 cell numbers. FGF10 drives cell protrusion by stimulating the ERK1/2 pathway and Arp2/3 based actin rearrangement. Developing AT2 cells are protected from the pro-AT1 effects of airway dilation and stretching by constricting their apical membrane via non-muscle myosin activity. Consistent with this model of FGF10-mediated AT2 cell differentiation, mice heterozygous for FGF10 or lacking FGFR2 have fewer protruding cells and subsequently reduced AT2 numbers.

## Role of FGF10 in Human Disease

### COPD and Developmental Airway Abnormalities

Early events in lung morphogenesis that impact formation of the conducting airways can have lifelong consequences. Defects in the formation of the trachea, bronchi, and bronchioles lead to increased turbulent airflow, accumulation of inhaled particulates, and reduced surface area available for gas exchange. Congenital defects in airway branching could cause disease in the immediate newborn period or in adulthood as defects in airway function predispose to chronic airway infection and injury. Decades ago, investigators proposed the concept of dysanaptic lung growth, where abnormalities in airway development could impact available surface area for gas exchange in the context of normal lung volume (Green et al., 1974). These defects become clinically significant during lung injury or challenges to normal gas exchange.

Chronic obstructive pulmonary disease (COPD) is a major cause of lung related illness and death throughout the world (Terzikhan et al., 2016). With aging populations, COPD now affects over 300 million people worldwide. While tobacco smoking clearly increases COPD risk, only approximately 20% of smokers develop COPD. In addition, between 20–45% of COPD patients are non-smokers but appear to develop airway disease through other environmental and occupational exposures (Salvi and Barnes, 2009). Recent studies connected genetic variants to structural airway abnormalities in patients with COPD (Smith et al., 2018). Up to 26% of the general human population have abnormalities in airway branching patterns, increasing COPD risk. Absence of the right medial-basal lung segment is present in approximately 6% of the population. These individuals have reduced luminal airway volume and a higher risk of COPD if also smokers. Investigators identified gene variants in the FGF10 intronic region in individuals with absence of the right medial-basal segment using two independent cohorts.

Striking results were also uncovered in a smaller study of adults with aplasia of salivary and lacrimal glands (ASLG), known to result from mutations causing FGF10 haploinsufficiency (Klar et al., 2011). Compared to both predicted values and unaffected siblings, subjects with FGF10 haploinsufficiency had significant, non-reversible airway obstruction by spirometry consistent with COPD. Similar studies have not yet been reported on patients with lacrimo-auriculo-dento-digital (LADD) syndrome, another disorder resulting from either FGF10 or FGFR2 haploinsufficiency (Shams et al., 2007). The rs1448044 SNP near the *FGF10* gene has been associated with reduced pulmonary function (Jackson et al., 2018), although the connection of this SNP to *FGF10* expression remains unclear. These studies provide strong evidence that mutations and variants in a critical gene during early lung morphogenesis (FGF10) can cause structural abnormalities in humans leading to disease following years of environmental exposures.

### Cystic Fibrosis, FGF10, and Airway Diameter

In addition to inherent defects in mucociliary clearance and bacterial killing, patients with cystic fibrosis (CF) have congenital abnormalities in airway shape and size (Meyerholz et al., 2010; Fischer et al., 2014). Infants with CF have smaller caliber tracheas and demonstrate airway abnormalities before developing clinical signs of respiratory infection (Sly et al., 2009). The developmental origins of these structural abnormalities have been confirmed and further investigated using the CF pig model. Newborn CF pigs have smaller diameter tracheas and bronchi compared to wild type pigs (Meyerholz et al., 2010). More recent studies measured reduced airway diameter in fetal CF pig lungs as early as the pseudoglandular stage of development during active branching morphogenesis of the conducting airways (Meyerholz et al., 2018). FGF10 treatment induced airway expansion in wild type fetal pig lung explants, but had no effect on CF explants. In the developing CF pig lung, FGF10 expression was similar to controls and could still increase epithelial proliferation in primary cultures of CF epithelia. However FGF10 could not stimulate CFTR-mediated increases in epithelial short circuit current in CF cells. So, while FGF10 signaling in CF lungs remains intact, the inability to stimulate epithelial transport impacts airway morphogenesis. The human and experimental animal data clearly show that early defects in fluid transport impact structural lung development in CF, likely contributing to the pathogenesis of CF lung disease.

### FGF10 and Connecting Inflammation to Newborn Lung Disease

Bronchopulmonary dysplasia (BPD) is the most common serious complication of extreme prematurity. Infants before 28-week gestation are still in the canalicular stage of lung development and must complete airway morphogenesis while exposed to the external environment. Infection and inflammation are the major clinical risk factors leading to BPD (Bhandari, 2014). In experimental models, fetal macrophage activation and IL-1β release inhibit saccular stage airway branching (Nold et al., 2013; Stouch et al., 2016). Reduction in saccular airway branching leads to fewer numbers of mature alveoli. In human newborn lungs, FGF10 localizes to clusters of cells within the lung interstitium. However in patients that died with BPD, fewer FGF10-positive cells could be detected throughout the lung tissue (Benjamin et al., 2007). The reduction in FGF10 could both have led to abnormal structural development and make the lung more susceptible to injury.

Inflammatory mediators that disrupt normal lung development in BPD do so at least in part by inhibiting FGF10 expression. Microbial products and inflammatory cytokines activate receptors that signal through the IKK/NF-κB pathway. Activated NF-κB translocates to the nucleus and regulates gene transcription (Dev et al., 2011). While mostly studied in the context of stimulating expression of pro-inflammatory response genes, NF-κB also inhibits expression of genes important for normal development. In the case of FGF10, NF-κB appears to interfere with the normal machinery that maintains FGF10 transcription in mesenchymal cells (Benjamin et al., 2007, 2010). Lacking a TATA sequence, the FGF10 promoter contains multiple conserved GC boxes which bind Sp family members and serve as sites of RNA polymerase recruitment (Carver et al., 2013). Sp1 binds the FGF10 promoter and stimulates transcription; Sp3 can either activate or repress FGF10 transcription. Upon nuclear translocation, NF-κB binds Sp3 and NF-κB-Sp3 complexes repress FGF10 promoter activity. Further understanding the molecular basis of FGF10 transcriptional regulation in normal and disease states could identify new treatment strategies for BPD and other clinical scenarios where maintaining FGF10 expression could provide benefit.

FGF10 is also critical for how the developing lung responds to injury. Mice heterozygous for FGF10 appear healthy with seemingly normal lung development. However haploinsufficiency for FGF10 leads to dramatic pathology in an experimental BPD model (Chao et al., 2017). Exposing newborn mice to transient hyperoxia leads to lung injury, inflammation, and reduced alveolarization. Wild type mice typically survive hyperoxia exposure with steady improvement in lung morphology. In contrast, FGF10 heterozygous mice exposed to hyperoxia following birth have more severe defects in alveolar development with 100% mortality. FGF10 heterozygous mice also have abnormal alveolar epithelial differentiation following hyperoxia with increased AT1 cells and reduced AT2 cell number. Reduction in AT2 cells could both impact surfactant production and lung compliance and prevent normal repair of alveolar structures following injury.

## Role of FGF10 in Airway Injury and Repair

Consistent with its role in airway morphogenesis, AT2 cell differentiation, and airway basal cell maintenance, FGF10 drives lung repair following various injuries. Pretreating adult rats with intratracheal FGF10 improves the recovery following high volume mechanical ventilation (Bi et al., 2014), altitude-associated hypoxia (She et al., 2012), bacterial endotoxin/sepsis (Tong et al., 2014), and ischemia/reperfusion (Fang et al., 2014). Most of these effects are thought due to increased AT2 expansion and/or differentiation. FGF10 also reduces bleomycin-induced lung fibrosis, potentially through its AT2 protective effects (Gupte et al., 2009). Large airway repair may involve a different mechanism. Following naphthalene treatment, activation of the basal cell niche in the trachea and large airways is required for epithelial repopulation and differentiation (Volckaert et al., 2017). Epithelial Myc and Yap activity stimulate mesenchymal FGF10 expression during the repair process, likely through Wnt ligands. Data in this study suggested overstimulation of this pathway might also lead to pathological airway changes.

## Rationale for Therapeutic Approaches

Because of its important roles in structural morphogenesis, epithelial differentiation, and protection from injury, FGF10 is an intriguing target for preventing and treating lung disease. Unfortunately, early human studies have so far failed to show a benefit of FGF10 in treating venous ulcers, mucositis, or ulcerative colitis (Sandborn et al., 2003; Plichta and Radek, 2012). As FGF10 is expressed by some cancer cell lines and can promote cell migration and proliferation, consideration of tumorigenic potential in preclinical studies will be critical (Sugimoto et al., 2014). Delivery of FGF10 into the correct biochemical HSPG containing microenvironment may represent a difficult therapeutic challenge. Compared to other FGF family members, FGF10 has unique HSPG binding characteristics with limited mobility when bound to the cell surface (Sun et al., 2016). In addition, the different relative activities of FGF10 and FGF7 may be due to their distinct HSPG interactions (Makarenkova et al., 2009). Other strategies could involve molecular and pharmacologic approaches aimed at increasing FGF10 expression and/or release. We now have multiple experimental animal lung disease models for testing these effects across the lifespan. Because constitutive overexpression of FGF10 during development can lead to dramatically altered lung morphogenesis (Gonzaga et al., 2008), the most effective approaches will likely promote the beneficial effects of FGF10 in the diseased lung while maintaining the complex regulatory mechanisms necessary for organ homeostasis.

## Author Contributions

LP surveyed the literature and wrote the manuscript.

## Conflict of Interest Statement

The author declares that the research was conducted in the absence of any commercial or financial relationships that could be construed as a potential conflict of interest.
